# *msbB *deletion confers acute sensitivity to CO_2 _in *Salmonella enterica *serovar Typhimurium that can be suppressed by a loss-of-function mutation in *zwf*

**DOI:** 10.1186/1471-2180-9-170

**Published:** 2009-08-18

**Authors:** Verena Karsten, Sean R Murray, Jeremy Pike, Kimberly Troy, Martina Ittensohn, Manvel Kondradzhyan, K Brooks Low, David Bermudes

**Affiliations:** 1 Vion Pharmaceuticals, Inc, New Haven, CT 06511, USA; 2 Biology Department, California State University Northridge, Northridge, CA 91330-8303, USA; 3 Center for Cancer and Developmental Biology, California State University Northridge, Northridge, CA 91330-8303, USA; 4 Alexion Pharmaceuticals, Inc, Cheshire, CT 06410, USA; 5 Ellington High School, Ellington, CT 06029, USA; 6 Yale University, Department of Therapeutic Radiology, New Haven, CT 06520, USA; 7 Aviex Technologies, LLC, Lake Peekskill, NY 10537, USA

## Abstract

**Background:**

Pathogens tolerate stress conditions that include low pH, oxidative stress, high salt and high temperature in order to survive inside and outside their hosts. Lipopolysaccharide (LPS), which forms the outer-leaflet of the outer membrane in Gram-negative bacteria, acts as a permeability barrier. The lipid A moiety of LPS anchors it to the outer membrane bilayer. The MsbB enzyme myristoylates the lipid A precursor and loss of this enzyme, in *Salmonella*, is correlated with reduced virulence and severe growth defects that can both be compensated with extragenic suppressor mutations.

**Results:**

We report here that *msbB *(or *msbB somA*) *Salmonella *are highly sensitive to physiological CO_2 _(5%), resulting in a 3-log reduction in plating efficiency. Under these conditions, *msbB Salmonella *form long filaments, bulge and lyse. These bacteria are also sensitive to acidic pH and high osmolarity. Although CO_2 _acidifies LB broth media, buffering LB to pH 7.5 did not restore growth of *msbB *mutants in CO_2_, indicating that the CO_2_-induced growth defects are not due to the effect of CO_2 _on the pH of the media. A transposon insertion in the glucose metabolism gene *zwf *compensates for the CO_2 _sensitivity of *msbB Salmonella*. The *msbB zwf *mutants grow on agar, or in broth, in the presence of 5% CO_2_. In addition, *msbB zwf *strains show improved growth in low pH or high osmolarity media compared to the single *msbB *mutant.

**Conclusion:**

These results demonstrate that *msbB *confers acute sensitivity to CO_2_, acidic pH, and high osmolarity. Disruption of *zwf *in *msbB *mutants restores growth in 5% CO_2 _and results in improved growth in acidic media or in media with high osmolarity. These results add to a growing list of phenotypes caused by *msbB *and mutations that suppress specific growth defects.

## Background

Lipopolysaccharide (LPS), the most abundant molecule on the surface of Gram-negative bacteria, acts as a permeability barrier and renders the outer-leaflet of the outer membrane (OM) relatively impermeable to hydrophobic antibiotics, detergents [1], and host complement [2]. LPS consists of three major components: lipid A, core polysaccharides and O-linked polysaccharides. Lipid A, with its fatty acid anchors [lauric, myristic and sometimes palmitic acid], is an endotoxin primarily responsible for TNFα-mediated septic shock. The addition of myristic acid to the lipid A precursor is catalyzed by the enzyme MsbB [3].

It has been shown that *msbB Salmonella *serovar Typhimurium exhibits severe growth defects in LB and sensitivity to bile salts (MacConkey) and EGTA-containing media. However, compensatory suppressor mutants can be isolated that grow under these conditions. One of these suppressor phenotypes results from a mutation in *somA*, a gene of unknown function [4]. *msbB Salmonella *Typhimurium strains have recently been developed as potential anti-cancer agents that possess impressive anti-tumor activity in mice [5]. In a phase I clinical study *msbB Salmonella *were shown to be safe in humans when administered *i.v*. However, bacteria were rapidly cleared from the peripheral blood of humans and targeting to human tumors was only observed in few patients at the highest dose levels of 3 × 10^8 ^CFU/m^2 ^and 1 × 10^9^/m^2 ^[6]. Toso et al. [6] noted that YS1646 (suppressed *msbB *strain, see below) grew best in air without added CO_2_.

The potential to grow in acidic and CO_2_-rich environments is a hallmark of pathogenic bacteria, enhancing persistence within phagocytes and survival inside the host. Sensitivity to CO_2 _and low pH of *msbB Salmonella *strains might explain poor colonization of tumors, which often contain high levels of CO_2 _and lactic acid [7,8] due to the Warburg effect, also known as aerobic glycolysis, whereby glucose uptake is elevated while oxidative phosphorylation is reduced, even in the presence of oxygen. Our previous work on suppressors of *msbB Salmonella *raised the possibility that secondary mutations could suppress sensitivity to 5% CO_2 _and acidic conditions.

Here we report that the growth of *msbB Salmonella *is highly inhibited (greater than 3-log reduction in plating efficiency) in a 5% CO_2 _atmosphere in LB media as well as under low pH conditions when compared to wild-type *Salmonella*. Furthermore, several CO_2 _resistant clones were selected from an *msbB Salmonella *transposon library (Tn*5*). Three mutations were mapped and all were shown to contain the Tn*5 *marker in the *zwf *gene, which encodes the enzyme glucose-6-phosphate-dehydrogenase and is tightly linked to the *msbB *gene.

## Results

### CO_2 _sensitivity of *msbB Salmonella*

**Figure 1:**
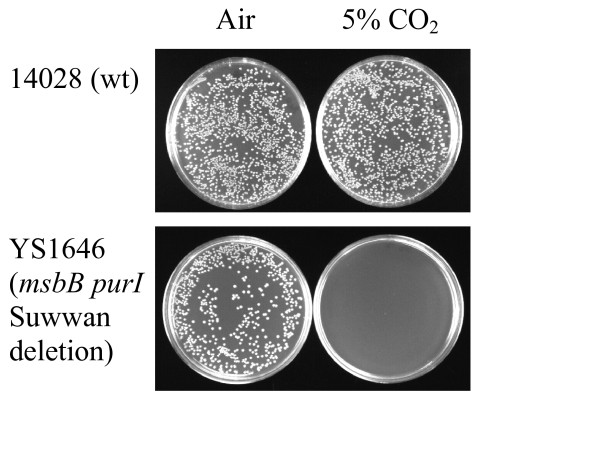
**Sensitivity and resistance to CO_2 _shown by comparing colony forming units (CFUs)**. Each strain was grown overnight in LB broth and diluted 10^6 ^fold, and then 100 μl was spread on each plate and incubated. Upper panel: wild type *Salmonella *(14028) on LB media in air (left) or 5% CO_2 _(right). Lower panel; YS1646 on LB media in air (left) or 5% CO_2 _(right).

CO_2 _sensitivity was first observed when YS1646, an *msbB purI *Suwwan deletion strain of *Salmonella *Typhimurium, was plated on blood or LB plates and incubated in a 5% CO_2 _incubator (Caroline Clairmont, personal communication; Toso et al., 2002). Suwwan deletion strains lack ~100 genes in the 17.7 to 19.9 Cs region of the chromosome [9]. In our studies, plating identical amounts (e.g., 100 μl of a 10^-5 ^dilution of a culture grown under non-selective conditions) to duplicate plates incubated at 37°C in either air or 5% CO_2_, few or no colonies of YS1646 were observed after 16 hours of incubation at 37°C in 5% CO_2 _(Figure 1). However, by plating more cells, the presence of a few resistant colonies could be detected, as we obtained 3.3 × 10^8 ^CFU/ml on plates incubated in air and 1.7 × 10^5 ^CFU/ml on plates incubated in the presence of 5% CO_2_, a greater than 3 log reduction. This CO_2 _sensitivity, first observed in YS1646, is also observed in a simple *msbB *mutant (see below). In contrast, wild-type *Salmonella *Typhimurium (ATCC 14028 and LT2), *Salmonella *Typhi (CS029, ATCC 33458), and *Escherichia coli *(MG1655, near-wild type K-12) are resistant to 5% CO_2 _(ATCC 14028: Figure 1; other strains: data not shown). Interestingly, *msbB E. coli *(KL423) was not sensitive to CO_2 _(not shown), consistent with there being physiologically relevant differences between the *E. coli *and *Salmonella *in regard to the loss of MsbB function, as has been previously observed [4]. These differences obscure or compensate for obvious growth defects in *msbB E. coli*.

CO_2 _sensitivity was found in all *msbB Salmonella *strains tested so far, indicating that CO_2 _sensitivity is a direct result of the lack of lipid A myristoylation (data not shown, see list of strains in Table 1). Consistent with these results, normal growth in CO_2 _was completely restored when *msbB *was expressed from a plasmid (pSM21(*msbB*^+^)) (see Table 1).

**Table 1:** Bacterial strains and plasmids

Strain or plasmid	Parental strain	Genotype	Derivation or source
*S. enterica *serovar Typhimurium			

14028	14028	Wild type	ATCC 14028

14028Δ*zwf*	14028	Δ*zwf82*	Replacement of *zwf *gene with Δ*zwf82 *by homologous recombination

14028 *gnd*	14028	*gnd-189*::MudJ (Kan^R^)	P22·DM4483 × 14028 → Kan_40_^R^

YS1646(=VNP20009)	14028	Δ*msbB2 *Δ*purI *ΔSuwwan	[9,31]

VNP20057	YS1646	Δ*msbB2 *Δ*purI zwf81*::Tn*5 *(Kan^R^) ΔSuwwan	YS1646 × P22 Tn5 pool (on 14028) → selection on LB plates in 5% CO_2_

YS1	14028	*msbB1*::Ωtet	[4]

YS1 *msbB*^+^	YS1	*msbB1*::Ωtet/pSM21*msbB*^+ ^(Amp^R^)	Plasmid pSM21 [4] into YS1

YS1 *zwf*(=YS11)	YS1	*msbB1*::Ωtet *zwf*:Tn*5 *(Kan^R^)	P22·VNP20057 × YS1 → Kan_20_^R^

YS873	14028	*msbB1*::Ωtet *somA1 zbj10*:Tn*10*	[4]

YS873 *msbB*^+ ^(=YS8731)	YS873	*msbB1*::Ωtet *somA1 zbj10*:Tn10/pSM21*msbB*^+ ^(Amp^R^)	Plasmid pSM21 [4] into YS873

YS873 *zwf*(=YS8732)	YS873	*msbB1*::Ωtet *somA1 zbj10*:Tn*10 zwf81*:Tn5 (Kan^R^)	P22·VNP20057 × YS873 → Kan_20_^R^

YS873 Δ*zwf *(=YS8733)	YS873	*msbB1*::Ωtet *somA1 zbj10*:Tn*10 Δzwf82*	Replacement of *zwf81*::Tn*5 *gene in YS873*zwf *with Δ*zwf82 *by homologous recombination

YS873 *gnd *(=YS8734)	YS873	*msbB1*::Ωtet *somA1 zbj10*:Tn*10 gnd-189*::MudJ (Kan^R^)	P22·DM4483 × YS873 → Kan_10_^R^

LT2	LT2	Wild type	ATCC 15277

DM4483	LT2	*gnd-189*::MudJ (Kan^R^)	Gift of Diana Downs and Eugene I. Vivas, U. of Wisconsin

YS501	LT2	*recD541*::Tn*10*dCm *hsdSA29 hsdSB121 hsdL6 metA22 metE551 trpC2 ilv-452 *H1-b H2-e,n,x *fla*-*66 nml*(-) *rpsL120 xyl*-*404 galE719*	[5]

*Salmonella enterica *serovar Typhi CS029			

*Salmonella enterica *serovar Typhi ATCC 33458			

*E. coli *K-12 MG1655	MG1655	F- l- *rph*-1	[32]

KL423	MG1655	F- l- *rph*-1 *msbB1*:: ΩCm	[4]

pCVD442		Amp^R^	[10]

pCVD442Δ*zwf*82		Amp^R^	This study

pSP72		Amp^R^	Promega Corporation

pSP72l*acZ*		*lacZ*, Amp^R^	This study

pSM21		*msbB*, Amp^R^	[4]

### The *somA *(for EGTA and salt resistance) and Suwwan deletion (for EGTA, salt, and galactose-MacConkey resistance) *msbB *suppressors do NOT suppress sensitivity to 5% CO_2_

Two *msbB Salmonella *strains with secondary mutations that allow faster growth are YS873 and YS1646. YS873 has a loss-of-function mutation in *somA *[4] and YS1646 has a large deletion, referred to as the Suwwan deletion [9], that includes *somA *plus ~100 other genes. The *somA *mutation in YS873 suppresses growth defects on EGTA and salt-containing media [4] and the Suwwan deletion in YS1646 suppresses sensitivity to EGTA, salt, and galactose MacConkey media [9]. However, neither the *somA *mutation nor the Suwwan deletion suppresses MsbB-mediated sensitivity to 5% CO_2 _(Suwwan deletion in YS1646, Figure 1; *somA *in YS873, see below). As shown in Figure 1, when plating identical dilutions containing greater than 100 CFU onto LB agar from an MSB broth culture of YS1646 and wild type *Salmonella*, no YS1646 colonies are detected after 24 hours of incubation in 5% CO_2 _at 37°C. Since we have not yet identified all of the genes within the Suwwan deletion that are responsible for the suppressor phenotype, we focused our study on YS873, which has clearly defined mutations in *msbB *and *somA*.

### CO_2 _resistant mutations are detected at high frequency in *msbB somA Salmonella*

Subsequent experiments revealed that spontaneous CO_2 _resistant mutants are detected when higher numbers of YS873 bacteria are plated and incubated under 5% CO_2 _conditions. The mutation frequency of spontaneous CO_2 _mutants from an MSB broth culture was determined to be ~3 out of 10^4 ^(not shown), which is similar to the frequency that EGTA and galactose MacConkey suppressor mutations arise in *msbB Salmonella *[4].

### A loss-of-function mutation in *zwf *suppresses CO_2 _sensitivity

**Figure 2:**
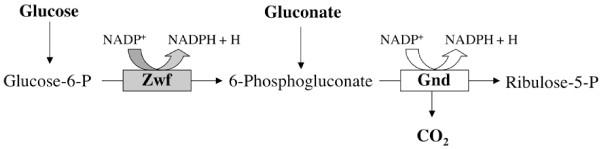
**Steps of the Pentose Phosphate Pathway (PPP) highlighting the relationship of the Zwf enzyme, gluconate, and Gnd-based production of CO_2_**.

In our preliminary studies, several spontaneous CO_2 _resistant mutants were isolated that showed a high degree of instability. Therefore, we subsequently focused on the use of Tn*5 *mutagenesis, which is known to generate stable insertions primarily associated with null mutations. To screen for a mutation that would compensate for CO_2 _sensitivity, a random Tn*5 *insertion library of YS1646 was created and selected on LB agar in 5% CO_2_. 9 clones were isolated, of which we determined the insertion sites in three of the clones using a genome-walking method. All of the Tn5 insertions identified were located in the monocistronic *zwf *gene. Two of the insertions (clones 14.2 and 32.2) were identical (possible siblings), located after open reading frame nucleotide 1019, and the third (clone 37.2) was located at after base pair 1349. Because we focused our screening on Tn*5 *insertions, we do not know if other mutagenesis methods would have isolated clones with mutations in other genes. *zwf *encodes glucose-6-phosphate-dehydrogenase, an enzyme of the pentose-phosphate-pathway (PPP). In this pathway, Zwf converts glucose-6-phosphate, from glycolysis, to 6-phosphogluconate, generating NADPH + H. The subsequent reaction, catalyzed by Gnd, converts 6-phosphogluconate to ribulose-5-phosphate, generating NADPH + H and CO_2 _(Figure 2). A non-polar deletion (see materials and methods) was created in *zwf *(Δ*zwf82*) using the pCVD442 vector [10] to test if the phenotypes arise from loss of the *zwf *gene or a polarity effect. The *zwf *non-polar deletion was found to exhibit the same CO_2 _growth phenotypes as the *zwf *Tn*5 *insertions. Subsequent experiments use the non-polar deletion in *zwf *in 14028 and YS873. A loss-of-function mutation in *zwf *results in smaller colony size than *zwf*^+ ^strains on agar media in both wild type and *msbB *genetic backgrounds.

### Gluconate prevents suppression of CO_2 _sensitivity by *zwf*

Zwf catalyzes the first step of the pentose phosphate pathway (PPP). PPP produces NADPH for anabolic pathways and the molecules generated by this pathway serve as building blocks for nucleotides, sugars, amino acids, and vitamins [11]. As shown in Figure 2, Zwf catalyzes the conversion of glucose-6-phosphate to 6-phosphogluconate. 6-phosphogluconate can also be formed from gluconate by gluconate kinase [12], which bypasses the PPP's requirement for Zwf (Figure 2). The addition of gluconate to media thereby allows for the production of 6-phosphogluconate in the absence of Zwf. The enzyme gluconate-6-phosphate dehydrogenase (Gnd) then decarboxylates 6-phosphogluconate, converting it from a 6-carbon to a 5-carbon (ribulose-5-phosphate) sugar and releasing CO_2 _gas. Perhaps a threshold of CO_2 _must be passed to inhibit the growth of *msbB Salmonella *and a loss-of-function mutation in *zwf *allows for the CO_2 _level to remain below this threshold. Previous reports of *zwf E. coli *show reduced CO_2 _production when grown in minimal media with acetate or pyruvate as a carbon source. However, *zwf E. coli *produced more CO_2 _than wild type when grown in minimal media with glucose [13,14]. Further studies will be required to clarify the production of CO_2 _by *Salmonella *grown in Luria-Bertani-based media and its contribution to CO_2 _sensitivity.

**Figure 3:**
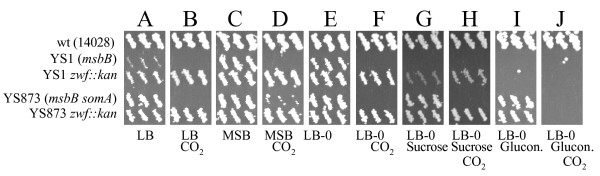
***zwf *mutation suppresses both *msbB*-induced CO_2 _sensitivity and osmotic defects**. Double velvet replica plates with different media were used to indicate the ability of small patches of bacteria (3 each) to grow. The strains used are listed on the left. Growth conditions (all at 37°C) included: A, LB media in air; B, LB media in 5% CO_2_; C, MSB media in air; D, MSB media in 5% CO_2_; E, LB-0 media in air; F, LB-O media in 5% CO_2_; G, LB-0 media containing sucrose (total 455 miliosmoles) in air; H, LB-0 media containing sucrose in 5% CO_2_; I, LB-0 + gluconate (glucon.) in air; J, LB-0 + gluconate in 5% CO_2_.

To test whether *zwf*'s suppressive effects result from its role in PPP pathway products and not from some unknown function, we observed the effect of gluconate on CO_2 _sensitivity in our mutants. Growth of YS873 *zwf *was tested on LB-0 plates containing 0.33% gluconate in ambient air and 5% CO_2 _(Figures 3I and 3J). As we hypothesized, YS873 *zwf *was not able to grow on LB-0 gluconate in 5% CO_2_. Thus, we confirmed that the *zwf*'s suppression of CO_2 _sensitivity results from its known enzymatic step in the PPP pathway. We also found a new phenotype for unsuppressed *msbB Salmonella*: YS1 does not grow on LB-0 agar in the presence of 0.33% gluconate (Figure 3I). To test if the production of 6-phosphogluconate or a downstream PPP metabolite is responsible for mediating CO_2 _resistance, we tested for CO_2 _resistance in a YS873 *gnd-189*::MudJ mutant (Gnd catalyzes the second step of the PPP pathway, Figure 2) and found that the strain remained CO_2 _sensitive (data not shown). Therefore, we conclude that the production of 6-phosphogluconate, by either Zwf or gluconate kinase, contributes to CO_2 _sensitivity in an *msbB *genetic background.

### *zwf *mutation suppresses both *msbB*-induced CO_2 _sensitivity and osmotic defects

For further analysis of the *msbB zwf *phenotype, the *zwf *(*zwf81*::Tn*5*) mutation was transduced into *msbB *(YS1) and *msbB somA *(YS873) genetic backgrounds to generate strains YS1 *zwf *and YS873 *zwf *respectively. As shown in the replica plate series of Figure 3, growth of unsuppressed YS1 is inhibited on LB (Figure 3A) and LB-0 gluconate (Figure 3I) but it grew well on MSB and LB-0 agar (Figures 3C and 3E), confirming the results of Murray et al. [4]. In contrast, growth of YS1 on MSB and LB-0 agar is completely inhibited when the plates are incubated in the presence of 5% CO_2_. The introduction of the *zwf *mutation completely compensates for the phenotype and allows the bacteria to grow under 5% CO_2 _on all three media (Figures 3B, 3D and 3F). However, it does not rescue YS1 from gluconate sensitivity (Figure 3I).

When NaCl in LB plates is substituted with sucrose at iso-osmotic concentrations (Figures 3G), growth of YS1 is also inhibited, indicating osmosensitivity of YS1. Interestingly, introduction of the *zwf *mutation improves growth of YS1 on LB and on LB-0 5% sucrose agar, indicating that the *zwf *mutation can partially compensate for the *msbB*-induced osmotic growth defect.

MSB media contains high levels of divalent cations, which have been proposed to increase lateral interactions between the phosphate groups of neighboring lipid A molecules [15]. Based on Murray et al.'s finding [16] that a decrease in electrostatic repulsion between the phosphates of lipid A can help to compensate for the lack of the myristic acid residue, we investigated whether Mg^2+ ^and Ca^2+ ^would protect against the detrimental effects of 5% CO_2_. On agar plates, Mg^2+ ^and Ca^2+^showed partial protection in YS873 (Figure 3D).

YS873, which contains the EGTA and salt resistance suppressor mutation *somA *[4], grows well on LB (Figure 3A), MSB (Figure 3C), LB-0 (Figure 3E) and LB-0 sucrose (Figure 3G) agar plates in air, but not when the plates are incubated in 5% CO_2 _(Figures 3B, 3D, 3F, and 3H). In contrast, the strain YS873 *zwf *is able to grow on all of these media in CO_2_, indicating that the *zwf *mutation can compensate for the growth defect of *msbB *strains in CO_2 _(Figure 3). Subsequent experiments were performed using the YS873 (*msbB somA*) genetic background because unsuppressed *msbB Salmonella *can not grow under mammalian physiological salt conditions [4].

### *msbB somA Salmonella *are sensitive to CO_2 _in LB and LB-0 broth

**Figure 4:**
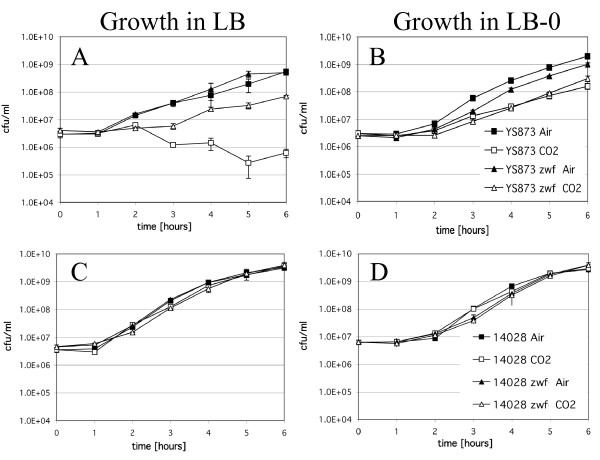
***msbB* confers growth sensitivity in liquid media under CO_2_ conditions containing physiological amounts of salt and this is suppressed by *zwf***. Two sets of *Salmonella *strains (YS873 and YS873 *zwf*; 14028 and 14028 *zwf*) were grown on either LB (A and C) or LB-0 (B and D) in either air or 5% CO_2_.

Figure 4 shows the growth of wild type ATCC 14028, 14028 *zwf*, YS873, and YS873 *zwf *in LB and LB-0 broth, incubated in the presence or absence of 5% CO_2_. As shown in Figure 4, the growth of YS873 (Figure 4A), but not ATCC 14028 (Figure 4C) is greatly impaired in LB broth in the presence of 5% CO_2_. A significant decrease in CFU is observed (Figure 4A), indicating that YS873 cells lose viability in the presence of 5% CO_2 _in LB broth. When a loss-of-function mutation in *zwf *is incorporated into YS873, no loss in viability is observed under identical conditions, although there is a longer lag phase of growth (Figure 4A). In LB-0 broth, there are no growth defects in 14028 or 14028 *zwf *(Figure 4D). For YS873 and YS873 *zwf*, the growth defects in LB-0 in the presence of 5% CO_2 _are attenuated in comparison to those observed in LB broth. There is no decrease in viability in YS873 in LB-0 in 5% CO_2_, although there is impaired growth in both YS873 and YS873 *zwf *in LB-0 in the presence of CO_2 _compared to growth in the absence of CO_2 _(Figure 4B).

### YS873 has severe morphological defects in LB broth under 5% CO_2 _conditions that are suppressed by a loss-of-function mutation in *zwf*

**Figure 5:**
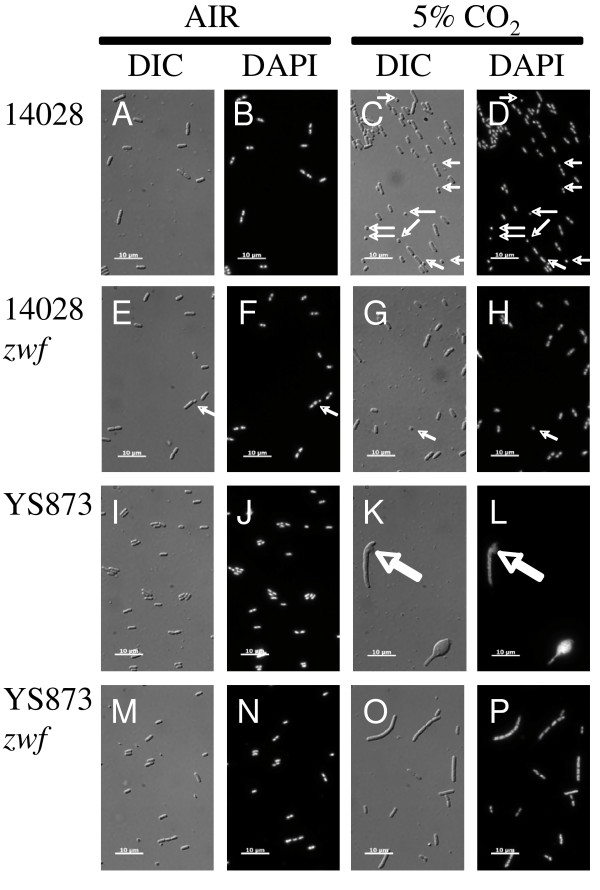
**YS873 has severe morphological defects in LB broth under 5% CO_2 _conditions that are suppressed by a loss-of-function mutation in *zwf***. DIC, Differential Interference Contrast; DAPI, 4'6-diamidino-2-phenylindole (DNA stain); Thick arrows point to lysis; Thin arrows point to mini-cells.

Since our results show that *msbB Salmonella *lose viability in the presence of 5% CO_2 _(Figure 4), we examined *msbB *mutants grown in the presence of 5% CO_2 _to determine if there are any defects in cell morphology or chromosome segregation. Differential interference contrast (DIC) microscopy shows striking morphological defects under CO_2 _conditions (Figure 5K), with long, bulging filamentous YS873 cells. DAPI staining shows no apparent chromosomal segregation defects, as no cells lacking DNA were observed (Figure 5L). However, the cell directly under the "K" and "L" labels appears to be lysing (see thick arrow).

As shown in Figures 5O and 5P, *zwf *suppresses the severe morphological defects in YS873 grown in LB in the presence of 5% CO_2_. Many cells are elongated but lack gross morphological defects. Growth in LB in a 5% CO_2 _environment caused wild type ATCC 14028 *Salmonella *to form minicells, with minicells (see thin arrows) accounting for ~15% of the cells (21/144) (Figure 5C and 5D as compared to Figures 5A and 5B). As seen in Figure 5E and 5F, 14028 *zwf *exhibits ~21% minicell formation in LB broth, even without CO_2 _(20/95 cells). Thus, we conclude that both CO_2 _and Zwf can, either directly or indirectly, affect cell division.

### β-galactosidase assays confirm cell lysis in LB in the presence of 5% CO_2_

**Figure 6:**
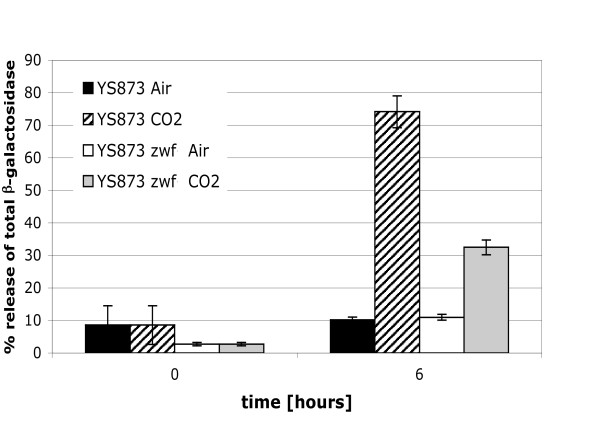
**β-galactosidase release assays confirm cell lysis in LB in the presence of 5% CO_2 _and that *zwf *confers resistance**. Release of β-galactosidase from the cytosol of the bacteria was used to test if the decrease in CFU observed in YS873, in LB in the presence of 5% CO_2_, resulted from cell lysis. The strains were grown under either ambient air or 5% CO_2 _conditions.

Microscopy (Figure 5K and 5L) suggested that some YS873 cells were lysing in LB in the presence of 5% CO_2_. To test if the decrease in CFU observed in YS873 in LB in the presence of 5% CO_2 _resulted from cell lysis, a plasmid expressing β-galactosidase was electroporated into YS873 and YS873 *zwf *and the cells were grown in LB in the presence or absence of CO_2_. As shown in Figure 6, after 6 hours of growth, significant cell lysis is observed in YS873 grown in the presence of 5% CO_2 _as measured by the release of the cytoplasmic enzyme β-galactosidase. Furthermore, a loss-of-function mutation in *zwf *significantly reduces cell lysis in YS873. No significant cell lysis is observed in the absence of CO_2_.

### CO_2 _sensitivity does not result from increased acidification of LB media and *zwf *suppresses sensitivity to acidic pH in LB broth

**Figure 7:**
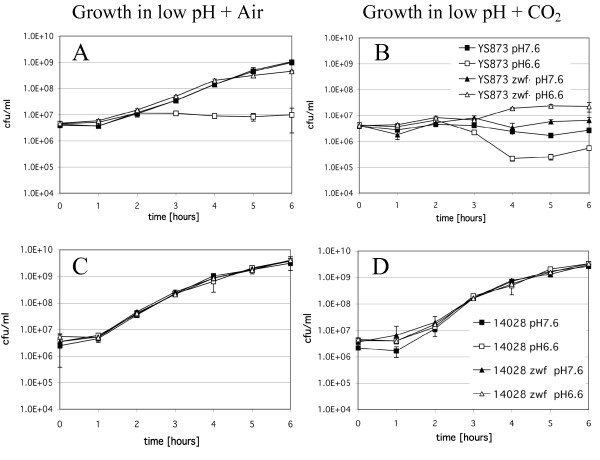
***zwf *suppresses sensitivity to acidic pH in LB broth in air, and to 5% CO_2 _in LB broth pH 6.6, but not pH 7.6**. Strains were grown in LB broth buffered to pH 6.6, or pH 7.6, in either air (A and C) or 5% CO_2 _(B and D).

During this study, we observed that the pH of LB broth dropped from pH 7.0 to pH 6.6 after equilibration in 5% CO_2_. Since CO_2 _can acidify bicarbonate buffered media, we tested whether part of the CO_2 _sensitivity was due to acidification of the media. Thus, to test if increased or decreased pH would alter sensitivity to CO_2 _in LB broth, we buffered LB broth to pH 7.6, or 6.6, and cultures were grown in the presence or absence of 5% CO_2_. As shown in Figure 7, wild type ATCC 14028 and ATCC 14028 *zwf *grow normally under all conditions in LB broth in the absence (Figure 7C) or presence (Figure 7D) of 5% CO_2_. In contrast, the growth of YS873 is significantly impaired when the pH of LB is 6.6, with no significant increase in CFU after 6 hours (Figure 7A), whereas when the pH of LB is 7.6, YS873 grows well (Figure 7A). A loss-of-function mutation in *zwf *allows for YS873 to grow well in LB broth at a pH of 6.6 (Figure 7A). 5% CO_2 _inhibited the growth of YS873 and YS873 *zwf *in LB pH 6.6 and 7.6 (Figure 7B). Although *zwf *protects against 5% CO_2 _in LB broth pH 6.6 (Fig 7B), it does not significantly improve survival in the presence of 5% CO_2 _in LB broth pH 7.6 (Figure 7B), suggesting that an acidic pH is a component for *zwf *to suppress *msbB*-mediated sensitivity to 5% CO_2_.

### β-galactosidase assays confirm cell lysis in LB broth, pH 6.6, in air

**Figure 8:**
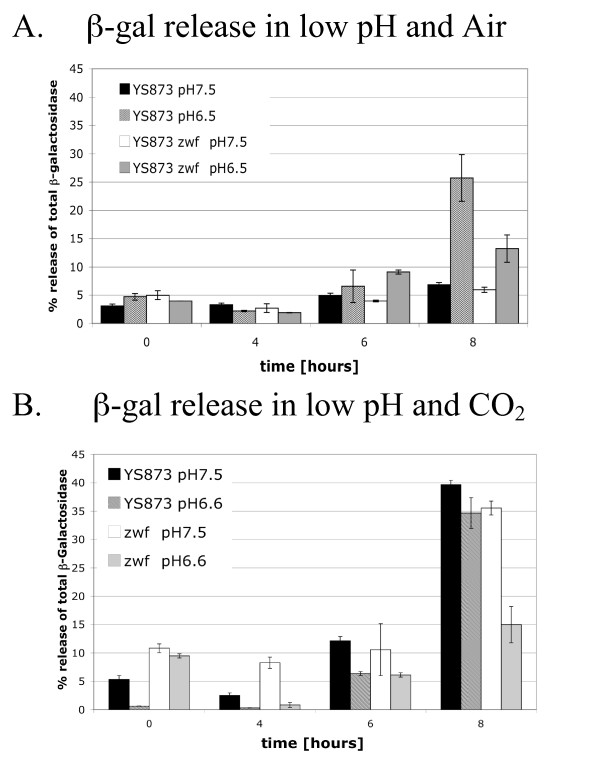
**β-galactosidase release assays confirm cell lysis in LB broth, pH 6.6, in air; *zwf *inhibits cell lysis in LB broth, pH 6.6, in air and in LB broth, pH 6.6, but not pH 7.6, in the presence of 5% CO_2_**. Release of β-galactosidase from the cytosol of the bacteria was used to test if the growth defects observed in YS873 and YS873 *zwf *resulted from cell lysis. Strains grown in LB broth at either pH 6.5, or pH 7.5, under either ambient air (A) or 5% CO_2 _(B) conditions.

To test if the loss of growth of YS873 in LB broth pH 6.6 was the result of cell death or simply the result of inhibition or delay of cell division, β-galactosidase release was measured. As shown in Figure 8A, significant cell lysis occurs after growth of YS873 for 8 hours in LB broth, pH 6.5 but not pH 7.5 (pH shifted slightly [+/-0.1 units] during autoclaving). Furthermore, a loss-of-function mutation in *zwf *significantly reduces cell lysis of YS873 grown in LB broth pH 6.5. This reduction in cell lysis, as measured by release of the cytoplasmic enzyme β-galactosidase, correlates with increased CFU/ml numbers observed in YS873 *zwf *(as compared to YS873) grown in LB broth, pH 6.6 (Figure 7A).

### *zwf *reduces YS873 cell lysis in the presence of 5% CO_2 _in LB broth pH 6.6, but not pH 7.6

Since we observed that YS873 lysed when there was no net growth in LB broth pH 6.5 while maintaining a relatively constant CFU/ml, we investigated if cell lysis occurs in YS873 *zwf*, which also exhibits little net growth with a relatively constant CFU/ml in the presence of 5% CO_2 _in LB broth pH 6.6 or 7.5 (Figure 7B). Growth curves for these strains indicated that there was a decrease in CFU/ml when YS873 was grown in LB broth pH 6.6 in the presence of 5% CO_2_, but that CFU/ml remained relatively constant if a loss-of-function mutation in *zwf *was present or if the pH of LB broth was 7.5 (Figure 7B). Figure 8 (8 hours) shows that significant cell lysis, as indicated by release of the cytoplasmic enzyme β-galactosidase, occurs when YS873 is grown in the presence of 5% CO_2 _at pH 6.6 or 7.6, and in YS873 *zwf *grown in the presence of 5% CO_2 _in LB pH 7.5. YS873 *zwf *exhibited significantly less lysis in the presence of 5% CO_2 _in LB broth pH 6.6, showing that a loss-of-function mutation in *zwf *significantly suppresses sensitivity to CO_2 _at neutral (as shown in Figure 6) or slightly acidic pH (Figure 8B). Again, we found that significant cell lysis can occur with a relatively constant CFU/ml (Figure 8B: YS873 *zwf *in LB pH 7.6).

## Discussion

### *msbB Salmonella *pleiotropy

The *msbB *gene was mutated to reduce the toxicity of *Salmonella *in mice and humans [5,6]. In order for these strains to function within mammalian systems they must be able to persist under normal mammalian physiological conditions. In contrast to other reports [17-20], we found *msbB Salmonella *to have striking growth defects, demonstrating sensitivity to salt, EGTA, MacConkey media, and polymyxin B sulfate [4,9,16]. Here we report additional sensitivity to osmolarity, gluconate, acidic pH and 5% CO_2 _growth conditions. Significantly, *msbB Salmonella *are sensitive to the conditions found within mammals, where blood has significant levels of salt and CO_2_; we therefore we screened for a suppressor of *msbB*-associated CO_2 _sensitivity.

### *zwf *supresses CO_2 _sensitivity in *msbB Salmonella*

Glucose-6-phosphate-dehdrogenase (encoded by *zwf*) catalyzes the first enzymatic step in the pentose phosphate pathway (PPP), which converts glucose-6-phosphate to 6-phosphogluconate and NADPH + H. In *E. coli*, *zwf *is regulated by several mechanisms including anaerobic growth [21], growth rate [22], weak acids as well as superoxide [23]. Weak acids appear to regulate *zwf *through the multiple antibiotic resistance (mar) regulon, whereas superoxide exposure induces *zwf *through the Sox R/S regulon and contributes to DNA repair [24]. *zwf *mutants of *Pseudomonas *are hypersensitive to superoxide generating agents such as methyl viologen [25].

*Salmonella *Typhimurium *zwf *might be regulated by a different set of environmental signals than *E. coli*. Superoxide, while clearly activating other SoxR/S regulated genes like *sodA *and *fumC*, does not induce *zwf *transcription [26]. *S*. Typhimurium *zwf *mutants have been shown to be less virulent in mice and more sensitive to reactive oxygen and nitrogen intermediates [27]. In general, it is thought that the expression of *zwf *and subsequent generation of NADPH helps cells to combat oxidative stress. Interestingly, SoxS mutants of *Salmonella *are not attenuated in mice [28], suggesting that even though *zwf *expression is important for survival, superoxide generated responses might not be required. In the case of *msbB *mutants, the *zwf *mutation restores wild type growth under 5% CO_2 _and pH 6.5 conditions, suggesting that the expression of *zwf *is detrimental for growth of *msbB *mutants in an acidic or increased CO_2 _atmosphere. Furthermore, our data showing that a loss-of-function mutation in *gnd *(which produces the second enzyme of the PPP pathway, Figure 2) does not suppress sensitivity to CO_2 _suggests that the production of 6-phosphogluconate, by either Zwf or gluconate kinase, contributes to CO_2 _sensitivity in *msbB Salmonella*.

### MsbB as a virulence factor?

Several publications cite MsbB as a virulence factor that is necessary for both septic shock and the ability to invade and persist in mammalian cells [5,17,29]. However, owing to the fact that *msbB Salmonella *were tested under 5% CO_2 _conditions, the lack of virulence may be partially or fully due to the inability of *msbB Salmonella *to grow in the presence of the 5% CO_2_. Further experimentation with *msbB zwf Salmonella *will be necessary to determine which virulence defects are attributable to *msbB *lipid A and those that arise from sensitivity to 5% CO_2_. Based upon this study and earlier studies on the sensitivity of *zwf *mutant to superoxides, *zwf *may both reduce virulence on one hand, yet potentiate growth under CO_2 _conditions on the other, further complicating virulence analyses.

## Conclusion

Here, we report new growth defects in *msbB Salmonella*: sensitivity to gluconate and growth in hypertonic, acidic or 5% CO_2 _conditions. These characteristics are in addition to the previously reported growth defects in the presence of salt, EGTA, polymyxin, or MacConkey media. Previous studies showing that MsbB is a virulence factor require further evaluation of the role that CO_2 _sensitivity plays. The potential for cryptic, spontaneous mutations remains a possibility that should be addressed by re-transduction under non-selective conditions followed by plating independently under CO_2 _and ambient air. We have created an *msbB somA zwf Salmonella *strain that is resistant to growth under acidic or 5% CO_2 _conditions. This strain contains a loss-of-function mutation in *zwf*, an enzyme in the pentose phosphate pathway that produces CO_2 _as it converts a 6 carbon sugar to a 5 carbon sugar. The study of the virulence of *msbB zwf Salmonella *will allow the determination of what types of virulence are attributable to cells having an MsbB lipid A independent of sensitivity to 5% CO_2_, which is required for *in vitro *and *in vivo *virulence assays.

## Methods

### Bacterial strains, plasmids, phage and media

The bacterial strains and plasmids used in this study are listed in Table 1. The *Salmonella msbB *insertion/deletion for tetracycline resistance was described by Low et al. [5]. P22 mutant HT105/1*int201 *(obtained from the *Salmonella *Genetic Stock Center, Calgary, Canada) was used for *Salmonella *transductions. *Salmonella enterica *serovar Typhimurium strains were grown on LB-0 or MSB agar or in LB, LB-0, buffered LB or MSB broth. MSB media consists of LB (Luria-Bertani media, [30]) with no NaCl and supplemented with 2 mM MgSO4 and 2 mM CaCl_2_. LB-0 is LB media with no NaCl. Buffered LB pH 7.5 and pH 6.5 consisted of LB-0 with 100 mM NaPO_4 _adjusted to 455 mOsmol by adding NaCl. MSB broth and agar were used for the growth of strains under non-selective conditions. LB-0 agar was used when using selective antibiotics in transductions and transformations. Plates were solidified with 1.5% agar. LB-0 agar or MSB broth were supplemented as needed with ampicillin (100 μg/ml) or kanamycin (20 μg/ml). Antibiotics were added to LB-0 agar after cooling to 45 degrees Celsius.

### Restoring *msbB*^+ ^genotype

In order to confirm that the observed CO_2 _sensitivity results simply from knocking out MsbB function, wild type *msbB *was expressed from the *msbB *promoter using plasmid pSM21 [4]. Purified plasmids were transformed into electroporation-competent cells of strains YS1 and YS873.

### Growth Analysis

Phenotypes of strains were determined by replica plating. Master plates were made on either MSB or LB-0 agar. Replica plating was performed using a double velvet technique [4]. Replica plates were incubated for 16 hours at 37°C. To generate growth curves, 3 ml broth tubes were inoculated with single colonies and grown on a shaker overnight at 37°C in air. Cells were diluted 1:1000 or 1:500 (β-gal strains) in LB broth. Cells were held on ice until all inoculations were completed. Triplicate cultures were then placed in a 37°C shaker with 250 rpm in air or 5% CO_2_. O.D._600_ was measured every 60 minutes and dilutions of bacteria were plated onto MSB or LB agar plates to calculate the number of colony forming units (CFU) per ml.

### Microscopic Observation

Strains 14028, 14028 *zwf*, YS873 and YS873 *zwf *were grown for 6 hours, as described above for growth curves, at 200 RPM. The cells were then fixed for microscopy using a solution of 30 mM sodium phosphate buffer (pH 7.5) and 2.5% formaldehyde. Cell morphology was observed with a Zeiss Axiovision microscope using differential interference contrast settings and DNA was detected via DAPI fluorescence. Fixed cells were incubated with 2 μg/ml DAPI for 10 minutes in the dark and aliquoted onto a 1% agarose pad.

### Mutation Frequency Determination

A frozen stock of YS873 was streaked on MSB media and incubated overnight at 37°C to isolate individual clones. Triplicate 3 ml of LB broth were inoculated with independent YS873 colonies. They were grown at 37°C in a shaker over night. The tubes were then placed on ice and diluted in 0.9% saline. 10-6 and 10-4 dilutions were plated in duplicates onto LB agar and incubated in air and CO_2 _incubators respectively overnight at 37°C to calculate the number of CFU per ml.

### Transduction and Transformation

*Salmonella *P22 transductions were performed by the method of Davis et al. [30], except that LB-0 plates supplemented with the appropriate antibiotic were used. EGTA was not added to the antibiotic plates for transductions. A BioRad Gene Pulser was used for electroporation with the following settings: 2.5 kV, 1000 ohms and 25 μFD for transformation of YS1 and 1.7 kV, 186 ohms and 25 μFD were used for YS873, YS1646, and ATCC 14028 [4].

### Tn5 mutagenesis and mapping

A library of transposons in YS1646 was made using the EZ::TN <Kan-2> insertion kit from Epicentre (Madison, WI). Over 56,000 kanamycin resistant (KanR) clones of YS1646 were pooled. The pool was screened for mutation rate and auxotrophy for different biosynthetic pathways by replica plating onto minimal media and media containing various pools of amino acids and bases [30]. Following selection for CO_2 _resistance by plating dilutions to LB-Kan and incubating in 5% CO_2_, the colonies were again pooled and a P22 lysate was generated and transduced to a non-suppressed strain and purified for kanamycin resistance under non-CO_2 _conditions in order to separate spontaneous mutants from Tn*5*-based suppressors. Transposon-associated Tn*5 *insertions were identified by replica plating in air and CO_2_. Mapping of the insertion sites was performed by using the GenomeWalker™ kit (Clonetech, Mountain View, CA) according to the manufacture's instructions.

### Construction of non-polar deletion in *zwf*

A non-polar deletion in *zwf *was generated by constructing a pCVD442 vector capable of deleting the entire *zwf *coding region by homologous recombination with the *Salmonella *chromosome [10]. Primers for PCR were designed that would generate one product immediately upstream of the 5' ATG start codon and a separate product immediately downstream of the 3' stop codon of the *zwf *coding region. The two separate products could then be ligated sequentially into the pCVD442 vector. The primers were: *zwf-*5'-reverse: 5'-GTGTGAGCTCGTGGCTTCGCGCGCCAGCGG CGTTCCAGC-3' (with added *Sac*I), *zwf*-5'-forward: 5'-GTGTGCATGCGGGGGG CCATATAGGCCGGGGATTTAAATGTCATTCTCCTTAGTTAATCTCCTGG-3' (with added *Sph*I), *zwf*-3' reverse: 5'-GTGTGCATGCGGGGTTAATTAA GGGGGCGGCCGCATTTGCCACTCACTCTTAGGTGG-3', and *zwf*-3'-forward: 5'-GTGTGTCGACCCTCGCGCAGCGGCGCATCCGGATGC-3'). The primers also generate internal *Not*I, *Pac*I, *Sph*I, *Sfi*I, and *Swa*I in order to facilitate cloning of DNA fragments into the Δ*zwf *for stable chromosomal integration without antibiotic resistance. This vector is referred to as pCVD442-Δ*zwf*. The presence of the deletion, in Amp^S ^Suc^R ^colonies, was detected by PCR using the following primers:*zwf*-FL-forward: 5'-ATATTACTCCTGGCGACTGC-3' and *zwf*-FL-reverse: 5'-CGACAATACGCTGTGTTACG-3'. Wild type produces a 2,026 base pair product whereas the mutant produces a 608 base pair (bp) product, a difference of 1418 bp, which corresponds to the size of the *zwf *gene (1475 bp minus a 57 bp multiple cloning site that replaces the open reading frame).

### β-galactosidase Assay

For β-galactosidase expression, *lacZ *was cloned into the high copy vector pSP72 (Promega) in *E. coli*, transformed into *Salmonella *strains (via restriction defective *Salmonella *strain YS501 [31], and screened for bright blue colonies on LB agar containing 40 μg/ml X-gal. *lacZ *was cloned from *E. coli *K-12 MG1655 [32] obtained from the Yale *E. coli *Genetic Stock Center (New Haven, CT) by PCR using the primers BGF1 5'-GATCGGATCCATGACCATGATTACGGATTCACTGGC-3' and BGR1 5'-GATCAAGCTTTTATTTTTGACACCAGACCAACTGG-3'. The PCR product was cut with *Bam*HI and *Hind*III and cloned into the plasmid pSP72 (Promega, Madison, WI) which had been cut with the same enzymes, transformed into DH5α, and selected for bright blue colonies on LB-amp plates containing 40 μg/ml X-gal. The plasmid was subsequently transformed to the restriction minus methylation plus strain YS501 before transforming other *Salmonella *strains. β-gal assays were performed according to the instructions for the Galacto-*Star*™ chemiluminescent reporter gene assay system (Applied Biosystems, Bedford, Massachusetts). Briefly, 1 ml of bacterial culture expressing β-gal from pSP72*lacZ *was pelleted at 13,000 × g for 5 min. Supernatants were filtered through a 0.2 μm syringe filter and then assayed immediately or frozen at -80°C until assayed with no further processing. Cell pellets were quickly freeze-thawed and suspended in 50 μl or 200 μl B-PER™ bacterial cell lysis reagent (Pierce Chemical) containing 10 mg/ml lysozyme (Sigma). Bacteria were allowed to lyse for 10–20 min. at room temperature and were then placed on ice. All reagents and samples were allowed to adjust to room temperature before use. Filtered supernatants and bacterial lysates were diluted as needed in Galacto-*Star*™ Lysis Solution or assayed directly. β-gal standard curves were made by preparing recombinant β-gal (Sigma, 600 units/mg) to 4.3 mg/ml stock concentration in 1× PBS. The stock was diluted in Lysis Solution to prepare a standard curve of 100 ng/ml- 0.05 ng/ml in doubling dilutions. 20 μl of standard or sample was added to each well of a 96-well tissue culture plate. 100 μl of Galacto-*Star*™ Subtrate, diluted 1:50 in Reaction Buffer Diluent, was added to each well and the plate rotated gently to mix. The plate was incubated for 90 minutes at 25°C in the dark and then read for 1 second/well in an L-max™ plate luminometer (Molecular Devices). Sample light units/ml were compared to the standard curve and values converted to units β-gal/ml. Percent release of β-gal was determined by dividing units/ml supernatant by total units/ml (units/ml supernatant + units/ml pellet). All samples were assayed in triplicate.

## Authors' contributions

DB was responsible for the overall project concept and design. VK, SRM and DB designed and planned the experiments. VK, SRM, JP, KT, MI, MK, KBL and DB performed the experiments and analyzed the results. VK, SRM, KBL and DB wrote the manuscript. All authors read and approved the final manuscript.
